# Supplementary material for: Mass Spectrometry-Based Proteomic Discovery of Prognostic Biomarkers in Adrenal Cortical Carcinoma
Source: Cancers (Basel). 2021 Aug 2;13(15):3890. doi: 10.3390/cancers13153890 (PMC8345732; doi:10.3390/cancers13153890)
Supplement: Supplementary file 1 [file cancers-13-03890-s001.zip › cancers-1283746-supplementary/cancers-1283746-supplementary--final.pdf]

Supplementary Materials

Mass Spectrometry-Based Proteomic Discovery of Prognostic Biomarkers in Adrenal Cortical Carcinoma

Han Na Jang, Sun Joon Moon, Kyeong Cheon Jung, Sang Wan Kim, Hyeyoon Kim, Dohyun Han and Jung Hee Kim

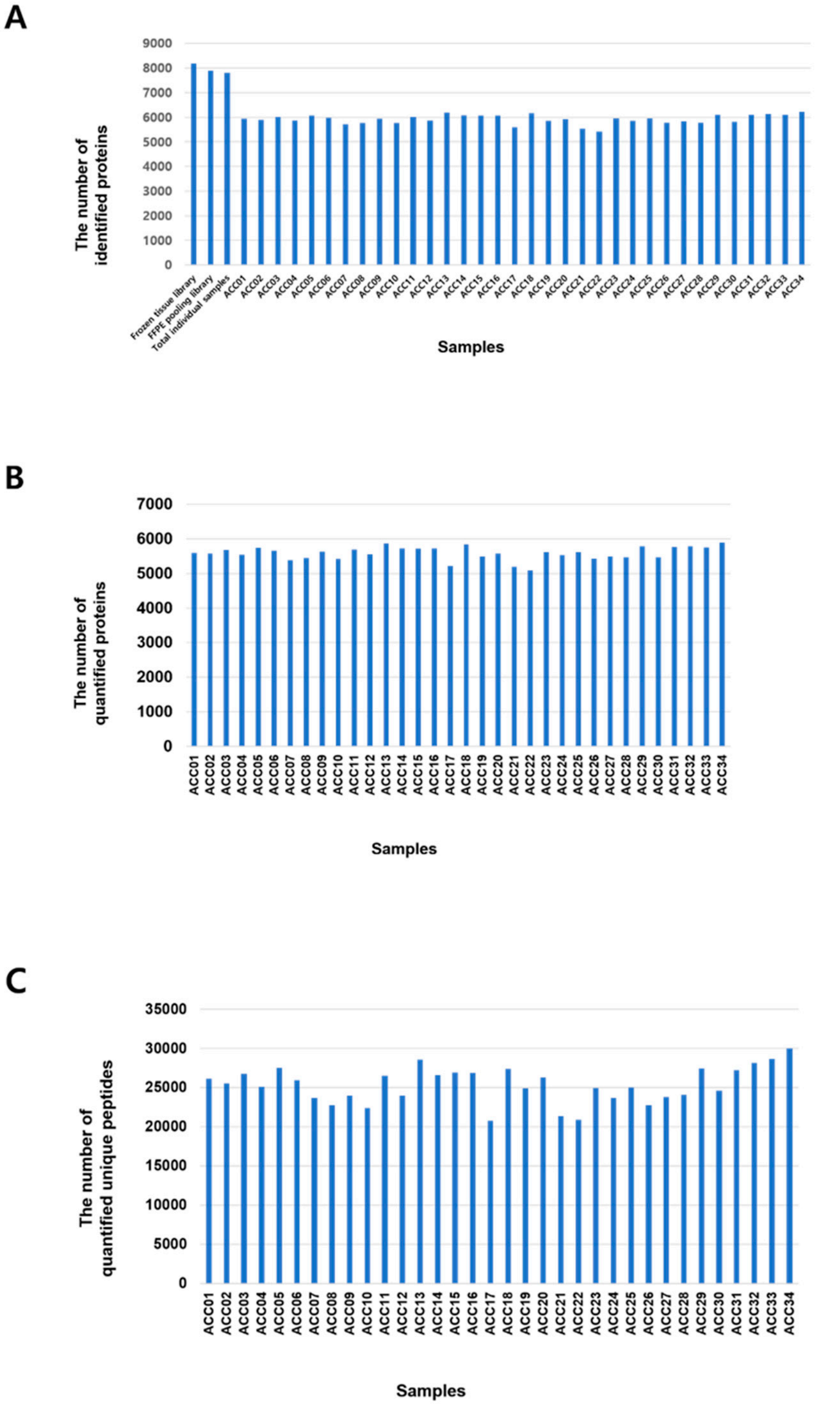

Figure S1. The number of proteins identified with a false discovery rate (FDR) < 1% at PSM and protein level. The numbers of identified proteins, quantified proteins, and quantified unique peptides in ACC tissues 1 to 34 were shown. ACC—adrenal cortical carcinoma.

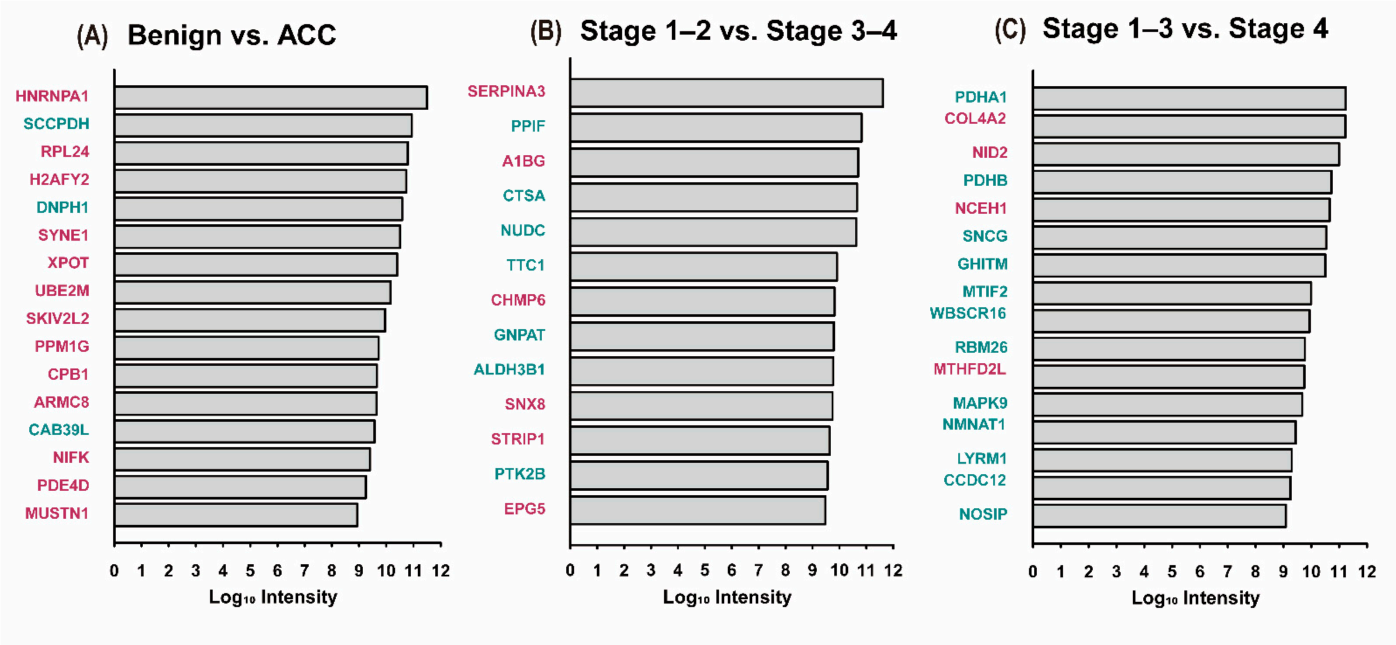

Figure S2. Signal intensity of feature selected proteins. The expression levels of feature-selected proteins obtained through ReliefF, infoGain, and ANOVA are presented. (**A**) Feature-selected proteins identified in adrenal adenoma are indicated in green, while proteins identified in ACC are indicated in red. (**B**) Feature-selected proteins identified in stages 1–2 ACC are indicated in green, while proteins identified in stages 3–4 ACC are indicated in red. (**C**) Feature-selected proteins identified in stages 1–3 ACC are indicated in green, while proteins identified in stage 4 ACC are indicated in red. A1BG—alpha-1-B glycoprotein; ACC—adrenal cortical carcinoma; ALDH3B1—aldehyde dehydrogenase 3 family member B1; ARMC8—armadillo repeat containing 8; CAB39L—calcium binding protein 39 like; CCDC12—coiled-coil domain containing 12; CHMP6—charged multivesicular body protein 6; COL4A2—collagen type IV alpha 2 chain; CPB1—carboxypeptidase B1; CTSA—cathepsin A; DNPH1—2'-deoxynucleoside 5'-phosphate N-hydrolase 1; EPG5—ectopic P-granules autophagy protein 5 homolog; GHITM—growth hormone inducible transmembrane protein; GNPAT—glyceronephosphate O-acyltransferase; H2AFY2—H2A histone family member Y2; HNRNPA1—heterogeneous nuclear ribonucleoprotein A1; LYRM1—LYR motif containing 1; MAPK9—mitogen-activated protein kinase 9; MTHFD2L—methylenetetrahydrofolate dehydrogenase (NADP+ dependent) 2 like; MTIF2—mitochondrial translational initiation factor 2; MUSTN1—musculoskeletal, embryonic nuclear protein 1; NCEH1—neutral cholesterol ester hydrolase 1; NID2—nidogen 2; NIFK—nucleolar protein interacting with the FHA domain of MKI67; NMNAT1—nicotinamide nucleotide adenylyltransferase 1; NOSIP—nitric oxide synthase interacting protein; NUDC—nuclear distribution C, dynein complex regulator; PDE4D—phosphodiesterase 4D; PDHA1—pyruvate dehydrogenase E1 alpha 1 subunit; PDHB—pyruvate dehydrogenase E1 beta subunit; PPIF—peptidylprolyl isomerase F; PPM1G—protein phosphatase, Mg2+/Mn2+ dependent 1G; PTK2B—protein tyrosine kinase 2 beta; RBM26—RNA binding motif protein 26; RPL24—ribosomal protein L24; SCCPDH—saccharopine dehydrogenase; SERPINA3—serpin family A member 3; SKIV2L2—superkiller viralicidic activity 2-like 2; SNCG—synuclein gamma; SNX8—sorting nexin 8; STRIP1—striatin interacting protein 1; SYNE1—spectrin repeat containing nuclear envelope protein 1; TTC1—tetratricopeptide repeat domain 1; UBE2M—ubiquitin conjugating enzyme E2 M; WBSCR16—williams-Beuren syndrome chromosome region 16; XPOT—exportin for tRNA.

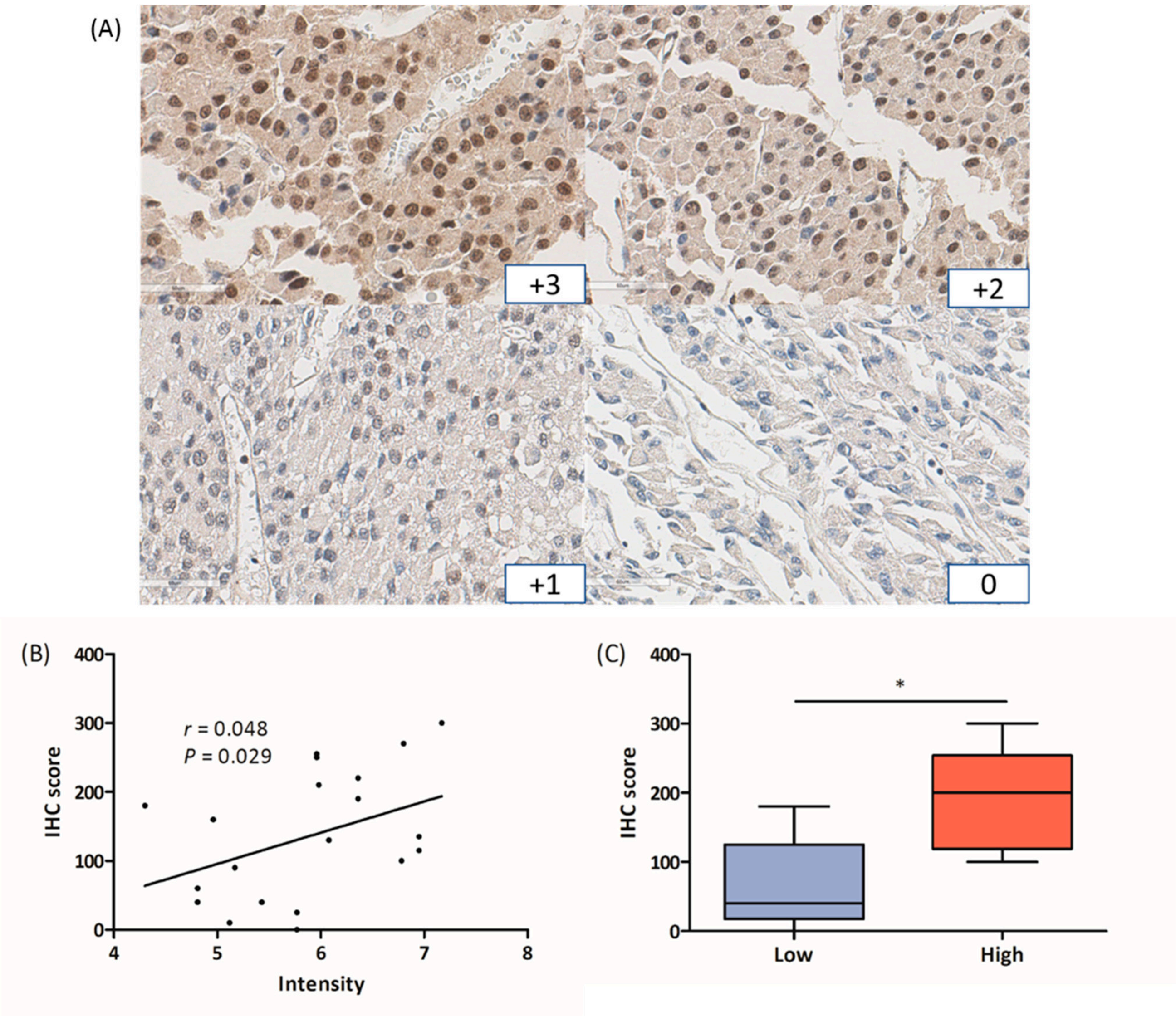

Figure S3. Immunohistochemistry staining of HNRNPA1 in ACC tissue, scatter plot between HNRNP1A expression intensity in proteomics and IHC score and difference in IHC score according to the expression level of HNRNP1A (**A**) Immunohistochemistry staining was performed with HNRNPA1, which is known to be associated with the poor prognosis of ACC in this study and also with poor prognosis in other tumors. Scoring was performed according to the degree of nuclear staining, and the area % corresponding to each score was indicated to calculate the IHC score. (**B**) The correlation between the expression level of HNRNP1A analyzed by mass spectrometry and the score in IHC staining was analyzed. There was a significant correlation between the two in Spearman's analysis ($r = 0.048$, $p = 0.029$). (**C**) Tissues were classified as low and high to the degree of expression of HNRNPA1 in mass spectrometry, and differences in IHC scores were analyzed. When Mann-Whitney analysis was performed, there was a significant difference in IHC score between the low expression group and the high expression group ($p = 0.002$). A significant difference in IHC score was indicated by * between the group with the low and high HNRNPA1 expression levels. ACC, adrenal cortical carcinoma; HNRNPA1—heterogeneous nuclear ribonucleoprotein A1; IHC, immunohistochemistry.

Table S2. IPA analyses for benign vs. ACC (Canonical pathway).

Pathway	$-\text{Log}_{10}\ p$	Z Score
Fatty Acid β-oxidation I	4.15	−1.414
Glioma Signaling	4.11	1.000
Sirtuin Signaling Pathway	3.84	1.706
Valine Degradation I	3.82	−2.449
Ethanol Degradation II	3.61	−1.890
Noradrenaline and Adrenaline Degradation	3.31	−2.646
Leucine Degradation I	3.23	−2.000
Serotonin Degradation	2.89	−1.667
Oxidative Phosphorylation	2.78	−1.941
Methylglyoxal Degradation III	2.55	−2.000
Apoptosis Signaling	2.53	1.155
Thrombopoietin Signaling	2.47	1.000
Isoleucine Degradation I	2.42	−2.000
ErbB4 Signaling	2.33	1.000
Superpathway of Citrulline Metabolism	2.30	2.000
Oxidative Ethanol Degradation III	2.30	−1.000
Tumoricidal Function of Hepatic Natural Killer Cells	2.27	2.000
Glycolysis I	2.27	2.236
TWEAK Signaling	2.25	1.633
α-Adrenergic Signaling	2.25	1.414
RhoA Signaling	2.20	−1.941
Insulin Receptor Signaling	2.20	−1.069
Granzyme B Signaling	2.19	2.000
Acute Phase Response Signaling	2.19	2.324
Glutathione-mediated Detoxification	2.19	−2.236
G Beta Gamma Signaling	2.17	1.387
Small Cell Lung Cancer Signaling	2.12	−1.342
VEGF Family Ligand-Receptor Interactions	2.11	1.265
Putrescine Degradation III	2.09	−2.000
Induction of Apoptosis by HIV1	2.07	1.134
NER Pathway	1.95	2.530
Tryptophan Degradation X (Mammalian, via Tryptamine)	1.91	−2.000
Ethanol Degradation IV	1.91	−1.000
Intrinsic Prothrombin Activation Pathway	1.85	1.633
EGF Signaling	1.77	1.890
Prolactin Signaling	1.76	1.000
Salvage Pathways of Pyrimidine Ribonucleotides	1.75	1.265
Ovarian Cancer Signaling	1.75	−1.134
UVA-Induced MAPK Signaling	1.70	−1.667
Glutathione Redox Reactions I	1.69	−1.000
Dopamine Degradation	1.62	−2.000
SAPK/JNK Signaling	1.59	1.265
NF-κB Signaling	1.58	1.291
TNFR1 Signaling	1.54	1.633
Gluconeogenesis I	1.50	2.000
Phospholipase C Signaling	1.48	1.500
Pyridoxal 5′-phosphate Salvage Pathway	1.44	1.134
Regulation of Cellular Mechanics by Calpain Protease	1.44	−2.000
Type I Diabetes Mellitus Signaling	1.44	1.633
ErbB Signaling	1.42	1.000
Production of Nitric Oxide and Reactive Oxygen Species in Macrophages	1.42	1.069
Neuregulin Signaling	1.39	1.000

Pathways with p values < 0.05 are presented and ordered by $\text{Log}_{10}\ p$ values. ACC—adrenal cortical carcinoma; EGF—epidermal growth factor; ErbB4—Erb-b2 receptor tyrosine kinase 4; ErbB—erythroblastic leukemia viral oncogene homolog; IPA—ingenuity pathway analysis; MAPK—mitogen-activated protein kinase; NER—nucleotide excision repair; NF-κB—nuclear factor kappa B ; SAPK/JNK—stress-activated protein kinase/c-Jun N-terminal kinase; TNFR1—tumor necrosis factor receptor 1; TWEAK—TNF-Like Weak Inducer of Apoptosis; UVA-ultraviolet A; VEGF—vascular endothelial growth factor.

Table S3. IPA analyses for stage 1–2 vs. stage 3–4 (Canonical pathway).

Pathway	Log_{10} *p*	Z Score
Acute Phase Response Signaling	22.30	2.524
LXR/RXR Activation	21.60	4.017
TCA Cycle II (Eukaryotic)	4.57	−2.449
Production of Nitric Oxide and Reactive Oxygen Species in Macrophages	4.55	3.873
GP6 Signaling Pathway	3.05	3.162
Valine Degradation I	2.99	−2.000
Superpathway of Methionine Degradation	1.90	−1.000
Inhibition of Matrix Metalloproteases	1.78	−1.000
Glioma Invasiveness Signaling	1.41	1.342
Apoptosis Signaling	1.37	−1.633

Pathways with *p* values < 0.05 are presented and ordered by Log_{10} *p* values. GP6—Glycoprotein VI; IPA, ingenuity pathway analysis; LXR/RXR—Liver X Receptor-Retinoid X Receptor; TCA—tricarboxylic acid cycle.

Table S4. IPA analyses for stage 1–3 vs. stage 4 (Canonical pathway).

Pathway	Log_{10} *p*	Z Score
Glutaryl-CoA Degradation	3.87	−2.000
Tryptophan Degradation III (Eukaryotic)	3.14	−2.000
tRNA Charging	3.04	−2.236
GP6 Signaling Pathway	2.63	2.121
Fatty Acid β-oxidation I	2.54	−2.000
Oxidative Phosphorylation	2.31	−1.890
Induction of Apoptosis by HIV1	2.16	−1.342
Leukocyte Extravasation Signaling	1.79	1.000

Pathways with *p* values < 0.05 are presented and ordered by Log_{10} *p* values. GP6—Glycoprotein VI; HIV1—human immunodeficiency virus-1; IPA, ingenuity pathway analysis.

Table S5. Baseline characteristics of TCGA study subjects.

Variables	ACC (*n* = 78)
Age	46.7 ± 15.8
Male	31 (39.7)
Initial Stage (ENSAT)	-
I	9 (11.5)
II	37 (47.4)
III	16 (20.5)
IV	14 (17.9)
Death	27 (34.6)
Follow-up, years (IQR)	3.3 (2.0–5.6)

There were 2 missing values in Stage. ACC, adrenal cortical carcinoma; ENSAT, European Network for the Study of Adrenal Tumor; IQR, interquartile range; TCGA, The Cancer Genome Atlas.

Table S6. Comparison of baseline characteristics of SNUH cohort and TCGA study.

Variables	ACC (*n* = 37)	ACC (*n* = 78)	*p*-Value
Age	48.5 ± 12.9	46.7 ± 15.8	0.663
Male	15 (40.5)	31 (39.7)	0.935
Initial Stage (ENSAT)	-	-	-
I	2 (5.4)	9 (11.5)	-
II	7 (18.9)	37 (47.4)	-
III	17 (45.9)	16 (20.5)	0.004
IV	11 (29.7)	14 (17.9)	-
Death	19 (51.4)	27 (34.6)	-
Follow-up, years (IQR)	4.0 (1.3–8.1)	3.3 (2.0–5.6)	0.596

ACC, adrenal cortical carcinoma; ENSAT, European Network for the Study of Adrenal Tumor; IQR, interquartile range; TCGA, The Cancer Genome Atlas.

© 2021 by the authors. Licensee MDPI, Basel, Switzerland. This article is an open access article distributed under the terms and conditions of the Creative Commons Attribution (CC BY) license (http://creativecommons.org/licenses/by/4.0/).
